# Infection with dengue-2 virus alters proteins in naturally expectorated saliva of *Aedes aegypti* mosquitoes

**DOI:** 10.1186/1756-3305-7-252

**Published:** 2014-05-30

**Authors:** Daniel M Chisenhall, Rebecca C Christofferson, Michael K McCracken, Ann-Marie F Johnson, Berlin Londono-Renteria, Christopher N Mores

**Affiliations:** 1 Department of Pathobiological Sciences, Vector-borne Disease Laboratories, Louisiana State University, School of Veterinary Medicine, Baton Rouge, LA, USA; 2 Center for Experimental Infectious Diseases, Louisiana State University, Baton Rouge, LA, USA

**Keywords:** Ae. aegypti, Dengue, Arbovirus infection, Transmission, Mosquito saliva, Salivary proteins, Transmission enhancement, Vectorial capacity modeling

## Abstract

**Background:**

Dengue virus (DENV) is responsible for up to approximately 300 million infections and an increasing number of deaths related to severe manifestations each year in affected countries throughout the tropics. It is critical to understand the drivers of this emergence, including the role of vector-virus interactions. When a DENV-infected *Aedes aegypti* mosquito bites a vertebrate, the virus is deposited along with a complex mixture of salivary proteins. However, the influence of a DENV infection upon the expectorated salivary proteome of its vector has yet to be determined.

**Methods:**

Therefore, we conducted a proteomic analysis using 2-D gel electrophoresis coupled with mass spectrometry based protein identification comparing the naturally expectorated saliva of *Aedes aegypti* infected with DENV-2 relative to that of uninfected *Aedes aegypti*.

**Results:**

Several proteins were found to be differentially expressed in the saliva of DENV-2 infected mosquitoes, in particular proteins with anti-hemostatic and pain inhibitory functions were significantly reduced. Hypothetical consequences of these particular protein reductions include increased biting rates and transmission success, and lead to alteration of transmission potential as calculated in our vectorial capacity model.

**Conclusions:**

We present our characterizations of these changes with regards to viral transmission and mosquito blood-feeding success. Further, we conclude that our proteomic analysis of *Aedes aegypti* saliva altered by DENV infection provides a unique opportunity to identify pro-viral impacts key to virus transmission.

## Background

Arboviral diseases are major burdens on the health of individuals and economies throughout the tropics and subtropics [1]. Dengue virus (DENV) is critically responsible for this impact, as it results in lost economic and academic productivity due to millions of cases of dengue fever, and it is the leading cause of childhood hospitalizations due to the severe manifestations of dengue hemorrhagic fever and dengue shock syndrome each year [2,3]. Due to the establishment of *Aedes aegypti* (*Ae. aegypti*) on the Portuguese island of Madeira, throughout the Black Sea coastal region, south Florida and several cities along the Gulf Coast in Texas and Louisiana; the potential for both DENV and *Ae. aegypti* to spread north as temperatures rise due to climate change is a serious threat [4-6]. Indeed, recently, autochthonous DENV transmission has been detected in Texas and Florida, as well as in France, Portugal, and Croatia [7-11].

In order to better characterize vector-viral interactions that might explain the expansion of DENV activity, several studies have determined the vector competence of *Ae. aegypti* with regards to DENV [12-14]. These determinations are very important because they have allowed researchers to parameterize the potential for transmission of various mosquito and viral combinations have, although the mechanisms behind observed differences remain elusive. Consequently, in order to characterize these interactions, some researchers have focused on exploring the effect a DENV infection has upon *Ae. aegypti* transcription, while others have focused on elucidating the impacts within an immune-response context [15-17]. Still other researchers have explored those interactions by massive computational efforts, such as *in silico* data analysis utilizing a systems biology approach [18]. While the body of information created in those efforts advance our understanding of the molecular events underpinning infection outcome within *Ae. aegypti*, characteristics of vector-pathogen interactions that directly impact DENV transmission requires detailed consideration of the impact of infection upon vector saliva.

When a DENV-infected *Ae. aegypti* bites a vertebrate, the virus is deposited along with a complex mixture of salivary proteins with diverse functions to facilitate blood-feeding. Those proteins are known to be anti-hemostatics, inhibitors of platelet aggregation, and anti-vasoconstricitves; along with allergens and immune-modulatory compounds [19-22]. Of particular importance to the virus-vector-vertebrate interface is the role the infection has on the salivary glands themselves, whose protein expression has been shown to be altered [23,24]. It may be that the DENV infection of *Ae. aegypti* salivary glands leads to an altered salivary expectorate, which when delivered to the bite site along with virus may enhance transmission success.

Accordingly, we investigated the ability of a DENV infection to change the quality of *Ae. aegypti* saliva. We characterized the proteins present in uninfected and DENV-infected mosquitoes and compared the relative abundance of matched proteins in each cohort. Herein, we describe these analyses and provide detailed consideration of the possible impacts DENV infection has upon its vector leading to transmission enhancement.

## Methods

### Virus

Dengue virus serotype-2 strain 1232 (DENV-2), originally isolated from a human patient in Jakarta, Indonesia in 1978 and provided by the World Reference Center of Emerging Viruses and Arboviruses, was previously passaged 6 times through African green monkey kidney (vero) cells before being used for this experiment. Subsequently, it was inoculated on vero cells grown at 37°C and 5% CO_2_ in Medium-199 with Earle’s salts, Penicillin/Streptomycin/Amphotericin B, and 10% fetal bovine serum [24]. After 5 days, the supernatant was harvested, titrated by plaque assay and qRT-PCR, and used at a concentration of 2.76x10^6^ plaque-forming units per mL [25].

### Mosquitoes

Laboratory strain *Ae. aegypti* (Rockefeller) were maintained under constant environmental conditions (28°C with a 16:8 light:dark photoperiod). The mosquitoes were allowed to feed on bovine blood in Alsever’s anticoagulant via Hemotek feeding device (Discovery Workshops, Lancashire, England), after which the blood-fed females were sorted and allowed to digest the blood meal for 4 days. Six cartons, each containing approximately 80 previously blood-fed females, were then intrathoracically-inoculated using an EntoSphynx Minucie (BioQuip, Rancho Dominguez, CA) dissecting needle dipped in viral stock. The control group of equal size (6 cartons of ~80 mosquitoes each) received an inoculation of media without virus.

### Saliva collection

After a 10-day extrinsic incubation period, mosquitoes were allowed to probe and feed on 1 mL of 1× phosphate-buffered saline (PBS) and 10 mM adenosine triphosphate (ATP) solution at 37°C in a Hemotek device for 1 hour; a variation on the technique developed by Ribeiro, Rossignol, and Spielman [19]. This was repeated after 72 hours to obtain a larger volume of PBS/ATP/saliva for downstream processing, approximately 12 mL. After the collection of saliva, the inoculated cohort was taken down and their legs were removed and placed in 900 μL of BA-1 diluent for viral RNA extraction and detection via qRT-PCR [26]. Disseminated infections were confirmed in >95% (n = 398/415) of the mosquitoes which supplied the ‘infected’ saliva solutions. From that dilute mixture, 200 μL fractions of the PBS/ATP/saliva solution were added to 800 μL of acetone chilled to −80°C and allowed to incubate at −20°C overnight. The tubes were then centrifuged at 4°C at 10,000 rpm for 10 minutes to pellet the precipitated protein. The supernatant was discarded and the resulting pellet was allowed to air-dry for 5 minutes at room temperature. Once translucent, the pellet was then reconstituted in 100 μL of 2-D rehydration buffer (Bio-Rad) consisting of: 8 M urea, 2% CHAPS, 50 mM dithiothreitol, 0.2% Bio-Lyte 3/10 ampholytes, and 0.001% bromophenol blue. The 100 μL of reconstituted protein solution from the first tube was then used to reconstitute the second pellet, further concentrating the protein solution. This serial reconstitution was performed no more than 5 times to minimize loss of recovery from over-saturation. The concentrated protein solution still contained too much salt for 1^st^ dimension focusing; therefore the samples were processed through a ReadyPrep 2-D cleanup kit (Bio-Rad) and resuspended again in 2-D rehydration buffer. Protein concentrations were determined with a NanoDrop spectrophotometer (Thermo Scientific).

### 2-D gel electrophoresis

30 μg of protein diluted in previously mentioned 2-D rehydration buffer to a final volume of 200 μL was loaded per sample per 11 cm, pH 3–10 nonlinear IPG strip (Bio-Rad) overlaid with approximately 2 mL of molecular biology-grade mineral oil (Bio-Rad) to prevent evaporation. First and second dimension electrophoresis conditions, gel staining, and imaging methods were performed as described previously [24].

### Image analysis

**Figure 1:**
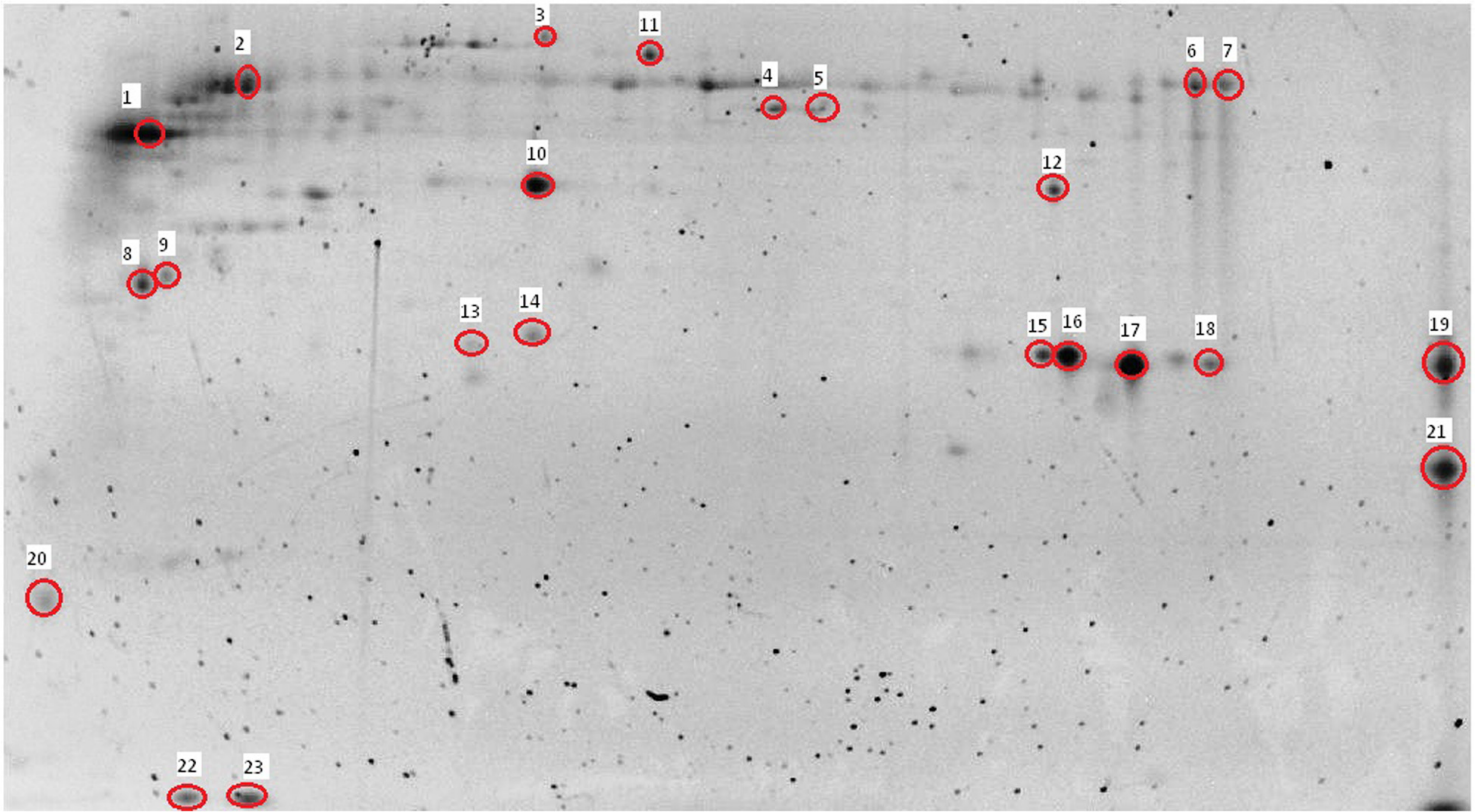
**Analyzed protein spots.** Representative *Ae. aegypti* saliva 2-D gel image (12.5% Tris–HCl in TGS buffer using a pH 3–10 non-linear IPG strip) with the spots that were cut circled and numbered to match the IDs in Table 1.

Two gels from each experimental condition (for a total of 4 gels) were analyzed to obtain both gel-to-gel differences between biological replicates and between experimental conditions, “infected saliva” and “non-infected saliva.” Pooling and sample size determination was performed according to acceptable practices in 2D SDS-PAGE and 2D DIGE proteomic analyses [24,27-31]. The normalized spot density values for both gels from each condition were used to determine the experimentally induced fold changes. Gels were normalized as previously described [24]. It is important to note that although ‘speckling’ did occur during staining and imaging, a known artifact associated with SYPRO® Ruby staining, the ‘speckle filter’ function of PDQuest was used to eliminate the small pixel intensity values from being included in the normalization calculations [32]. This allowed determination of the changes in protein spot abundance due to our experimental treatment. The relative expression levels of the individual spots were evaluated by calculating a fold-change value per spot as the infected spot intensity divided by the uninfected control spot intensity to determine the change relative to infection with DENV-2. A representative gel image has been provided with the spots excised for mass spectrometry analysis marked and numbered for reference (Figure 1).

### Mass spectrometry

After image analysis, a representative gel from each group containing all of the spots of interest was sent to the Nevada Proteomics Center at the University of Nevada, Reno for robotic spot excision, trypsin digestion and liquid chromatography-tandem mass spectrometry (LC-MS/MS) analysis utilizing a Thermo LTQ Orbitrap XL mass spectrometer with ETD coupled to a Michrom Paradigm MDLC and Michrom CaptiveSpray (Thermo Scientific). All MS/MS samples were analyzed using Sequest (Thermo Fisher Scientific, San Jose, CA, USA; version v.27, rev. 11). Sequest was set up to search an *Ae. aegypti* database (35667 entries) downloaded from Vectorbase.org and the non-redundant database ‘nr’ from NCBI for confirmation, assuming the digestion enzyme trypsin [33]. Sequest was searched with a fragment ion mass tolerance of 1.00 Da and a parent ion tolerance of 0.0068 Da to 0.041 Da, depending on the spot analyzed. Iodoacetamide derivative of cysteine was specified in Sequest as a fixed modification. Oxidation of methionine was specified in Sequest as a variable modification.

Scaffold (version Scaffold_3.4.3, Proteome Software Inc., Portland, OR) was used to validate MS/MS based peptide and protein identifications. Peptide identifications were accepted if they could be established at greater than 95.0% probability as specified by the Peptide Prophet algorithm [34]. Protein identifications were accepted if they could be established at greater than 95.0% probability and contained at least 2 identified peptides. Protein probabilities were assigned by the Protein Prophet algorithm [35]. Proteins that contained similar peptides and could not be differentiated based on MS/MS analysis alone were grouped to satisfy the principles of parsimony.

### Transmission modeling

To demonstrate a potential consequence of altered salivary quality upon transmission measures, two parameters were derived to account for the hypothetical difference in the biting rate of infected versus uninfected mosquitoes and enhancement of transmission success. Using the framework of vectorial capacity- a quantity that is used to estimate the number of secondary infectious bites resulting from a single, primary infectious bite- measures the force of infection of mosquito to human transmission [12,36]. The parameter values and vectorial equations are given in Additional file 1: SI1. To isolate the potential effects of the altered feeding environment (rendered by the reduction of pain-inhibitory and anti-hemostatic proteins in *Ae. aegypti* saliva), the daily biting rate of *uninfected* mosquitoes was set at **a = .63**[37], while the biting rate of *infected* mosquitoes was investigated over the range **a**_**INF **_**= [.63-1.63]**. Similarly, the altered probability of transmission success rendered by additional probing (and presumably deposition of virus into the skin) was investigated by adding the parameter **t**, which was varied from .7 to 1 [38]. The addition of this parameter is based on the knowledge that not all infectious bites result in a productive infection, and our hypothesis that additional feeding (through increased salivation or separate bites) increases the probability of such. The change in vectorial capacity was calculated by taking the difference between traditional vectorial capacity calculation (where a = a_INF_) and this new modified vectorial capacity (where a < a_INF_). The magnitude of the potential enhancement of transmission success was investigated over the range of **t** and the alteration of vectorial capacity expressed as the difference from t_min_ = .5 [38].

## Results

Seventy-four spots were matched across the 4 gels created from the cleaned-up, naturally-expectorated saliva. Of those 74 spots, 23 spots were chosen to be analyzed by mass spectrometry due to their being differentially expressed or present in very high quantities, which would be informative for land-marking purposes. The proteins identified within those 23 excised and analyzed spots were: a DEAD-box ATP-dependent RNA helicase, beta chain spectrin, the hypothetical secreted protein AAEL000748, adenosine deaminase, a putative adenosine deaminase, apyrase, a putative apyrase, an inosine-uridine preferring nucleoside hydrolase, a putative purine hydrolase, a salivary anti-FXa serpin, the hypothetical protein AAEL000732, a putative serine protease inhibitor (Serpin-4), an angiopoietin-like protein variant, a putative 34kD family secreted salivary protein, D7 (3 spots), a putative D7, a low density lipoprotein receptor, a putative 30kD secreted protein (‘short-form aegyptin’), a venom allergen/antigen 5, and a putative C-type lectin (2 spots). Their respective fold-changes in the infected saliva, along with accession numbers, are located in Table 1. The locations of these proteins in the gel can be found in Figure 1.

**Table 1:** Identified proteins with accession numbers and fold change information

***Ae. aegypti *salivary proteins identified by mass spectrometry with fold change due to DENV infection**			
**GenBank ID**	**Protein name**	**Spot**	**Spot fold change**
gi|108875535|	DEAD-Box ATP-Dependent RNA Helicase^‡^	1	−1.5
gi|108877982|	Beta Chain Spectrin^‡^	2	+1.0
gi|157109431|	Hypothetical Secreted Protein AAEL000748^‡^	3	−4.0
gi|108878609|	Adenosine Deaminase	4	−2.8*
gi|18568326|	Putative Adenosine Deaminase	5	−5.2*
gi|1094353|	Apyrase^‡^	6	−1.1
gi|108877845|	Putative Apyrase^‡^	7	+1.1
gi|108877687|	Inosine-Uridine Preferring Nucleoside Hydrolase	8	−7.9*
gi|18568280|	Putative Purine Hydrolase	9	−3.8*
gi|94468358|	Salivary Anti-FXa Serpin	10	−4.8*
gi|157109433|	Hypothetical Protein AAEL000732	11	−7.7*
gi|157131306|	Putative Serine Protease Inhibitor (Serpin-4)	12	−19.4*
gi|94468352|	Angiopoietin-Like Protein Variant^‡^	13	−1.8
gi|94468642|	Putative 34kD Family Secreted Salivary Protein^‡^	14	−1.3
gi|222447044|	D7^‡^	15	−4.5
gi|222447044|	D7^‡^	16	−1.3
gi|222447044|	D7^‡^	17	−1.8
gi|108877064|	Low Density Lipoprotein Receptor	18	−11.3*
gi|157113327|	Putative D7^‡^	19	−3.5
gi|18568322|	Putative 30kD Secreted Protein; ‘Short-Form Aegyptin’	20	−14.1*
gi|157110207|	Antigen-5/Venom Allergen^‡^	21	−2.5
gi|18568318|	Putative C-Type Lectin^‡^	22	N/A^1^
gi|18568318|	Putative C-Type Lectin^‡^	23	N/A^1^

*denotes significance at the p ≤ 0.05 level via Student’s t-test of the mean density of each spot between experimental conditions (n = 2 per condition).

^1^quantity at these spots were unable to be calculated due to the location of the bromophenol blue dye front in two of the four gels.

^‡^denotes proteins from spots chosen for mass spectrometry identification for the purposes of landmarking.

**Figure 2:**
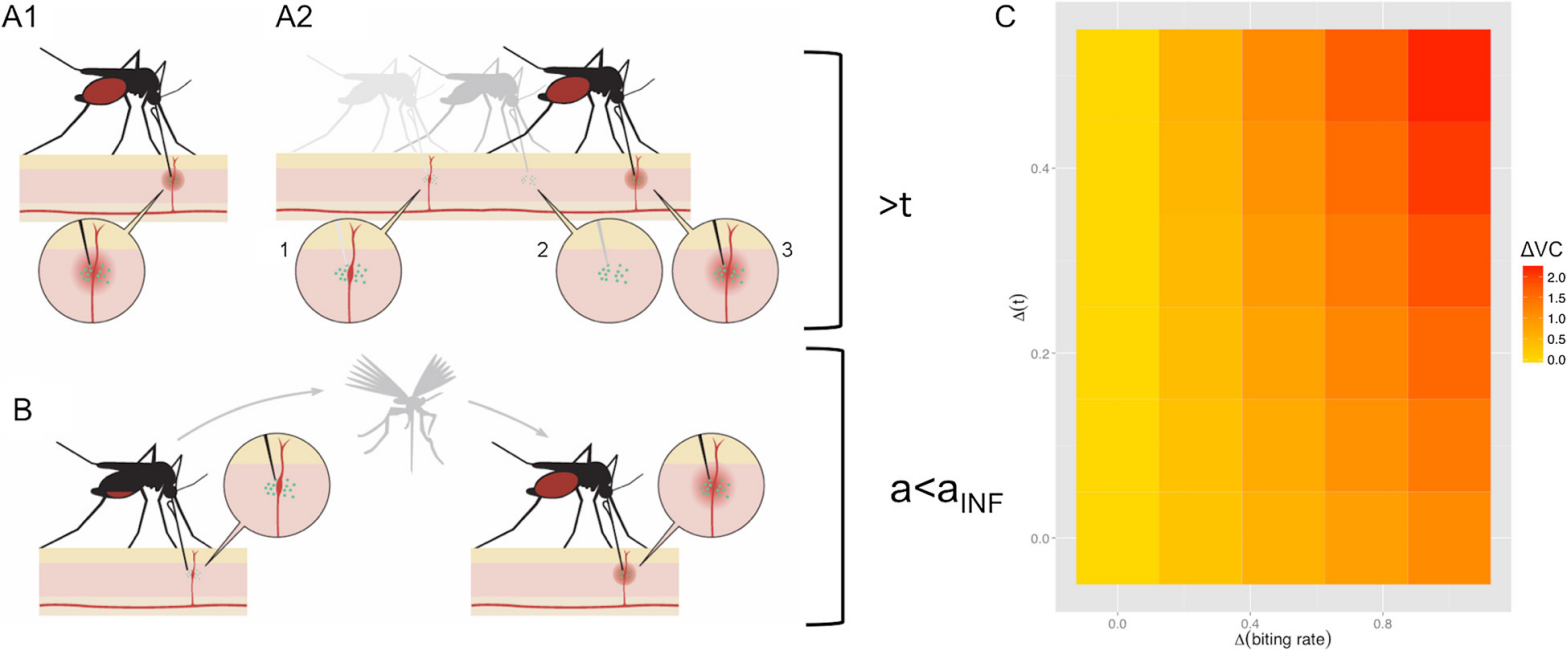
**The manifold potential effects of altered salivary proteins on mosquito feeding and DENV transmission.** In order to achieve a successful blood feeding, an infected mosquito might: **A1)** Increase its salivary, and consequentially viral, inoculum in order to restore a normal level of anti-hemostatic and pain-reducing salivary proteins relative to an uninfected mosquito; **A2)** given reduced anti-hemostatic and pain-reducing salivary proteins, attempt refeeding if (1) the pain perception at the bite site alerts the host or leads to a clot-induced disruption of feeding (2) causing the mosquito to seek another bite site, increasing overall viral inoculum (3) represented by increasing t (the probability of transmission success) in our vectorial capacity (VC) equation; **B)** Alternatively, if this interrupted female moved on to another host in order to acquire a sufficient blood-meal after a clot-induced or host-triggered interruption, then a subsequent transmission event could occur, even though DENV transmission had previously occurred during the failed previous feeding attempt, represented by a_INF_ in our VC equation. **C)** The impacts of these potential transmission enhancements due to changes in transmission success probability and daily biting rate could yield an increase in the vectorial capacity of the mosquito (ΔVC) relative to a baseline calculation of VC, and is represented by increasingly darker colors. The x-axis is the difference in probability of transmission success relative to baseline (t_min_ = .5) and the y-axis is the difference in in biting rate between uninfected mosquitoes and infected mosquitoes (a-a_INF_) = Δ(daily biting rate). Thus the coordinates (0,0) refer to t = .7 (Δ transmission success of .2) and a = a_INF_ (no enhancement to biting rate).

In addition, all mass spectrometry related data has been included in the Additional file 2– Mass_Spec_Supplement.To account for differences in the salivary proteins of infected mosquitoes involved in anti-hemostatic and pain responses at the bite site, modifications to the vectorial capacity equation were made. The biting rate, which is usually assumed to be the same for all mosquitoes, was estimated separately based on infection status. Accounting for the increase in biting rate of infected mosquitoes relative to uninfected mosquitoes resulted in a linear increase in vectorial capacity. The potential enhancement of transmission success had a similarly linear effect, though since this value is a probability, it is necessarily bounded by 1, and thus its impact is also bounded. Taken together, these changes in proteins could result in as many as two additional infectious bites previously unaccounted for in traditional parameterizations of vectorial capacity (Figure 2).

## Discussion

The majority of the protein identifications determined by mass spectrometry were for known or putative salivary proteins, a total of 16 out of 19 unique IDs [39,40]. The remaining three proteins with previously undetected salivary roles (the DEAD-box ATP-dependent RNA helicase, beta chain spectrin, and the low density lipoprotein receptor) could be present in this salivary sample due to normal cell death in the salivary gland, as has been seen previously [41]. Alternatively, these proteins may be being utilized in non-traditional roles to facilitate blood-feeding as kratagonists, proteins that scavenge host hemostatic or inflammatory system components [42,43]. For instance, the ATP-binding motif present in the DEAD-box ATP-dependent RNA helicase may be fortifying the function of other ATP-degrading salivary proteins like apyrase [39,44].

Likewise, the low density lipoprotein receptor (LDL-r) family has been shown to include members who are involved in a diverse array of cellular functions, including the LDL-r related protein (LRP)-type receptors which regulate proteolytic processes involved in fibrinolysis and coagulation [45]. It is interesting to note that in the saliva of DENV-2 infected *Ae. aegypti*, this protein’s expression was reduced 11.3 fold, which was statistically significant between treatment groups (p ≤ 0.05). In fact, the *Ae. aegypti* low density lipoprotein receptor (gi|108877064|) is more structurally similar to the mammalian LRP receptor than the archetypal mammalian LDL-r, according to the Conserved Domain Archetecture Retrieval Tool (cDART) through the National Center for Biotechnology Information (NCBI) [46]. This would suggest that the *Ae. aegypti* LDL-r may share similar binding properties of other LRP-type receptors and therefore could disrupt mammalian LRP-mediated proteolytic regulation in a kratagonistic fashion. Additionally, a secreted LRP receptor, referred to as sLRP-1 for ‘shed LRP-1’, has recently been found in human plasma, cerebrospinal fluid, and the brain [47]. This soluble receptor is produced from the full-length LRP-1 protein that is cleaved extracellularly in response to inflammation [48]. Further research has determined that injected sLRP-1 decreases inflammation and neuropathic pain in a mouse model [49]. Whether the *Ae. aegypti* salivary form of this protein deposited at the bite site is behaving the same way remains to be seen.

Another group of proteins whose expression was significantly decreased in DENV-2 infected saliva were the adenosine deaminases, the archetypal protein and a putative version, 2.8 fold and 5.2 fold, respectively (p ≤ 0.05). An amino acid sequence comparison between the two proteins reveals that the putative version shares 97% identity with adenosine deaminase and is slightly larger by 0.692 kDa, in agreement with the almost imperceptible migration difference in the 2-D gel. Adenosine deaminase has been shown to convert adenosine at the bite site into inosine and ammonia, which acts to prevent peripheral pain signaling [50]. Ribiero *et al.* noted that the reduction in adenosine at the bite site by adenosine deaminase was perplexing due to the fact that adenosine is a vasodialator and platlet aggregation inhibitor, functions that facilitate blood-feeding; therefore they concluded that the secondary characteristic of pain initiation must trump those functions for a diurnal-feeding mosquito such as *Ae. aegypti*.

Mosquito salivary nucleosidases, such as the inosine-uridine preferring nucleoside hydrolase (reduced 7.9 fold, p ≤ 0.05) and the purine hydrolase (reduced 3.8 fold, p ≤ 0.05) found during our mass spectrometry-based identification effort, convert nucleosides into D-ribose and their respective purine or pyrimidine base with the addition of water and occasionally the assistance of a divalent cation, such as calcium [51]. While the inosine-uridine preferring nucleoside hydrolase will hydrolyze both purines and pyrimidines, the purine hydrolase is specific for inosine, adenosine, and guanosine [52]. With the assistance of adenosine deaminase, any adenosine at the bite site would likely be converted into inosine and then subject to either of the two hydrolases above, leading to the complete removal of any mast cell degranulation-inducing products [53]. Given the synergy of these hydrolases and adenosine deaminase, it is interesting that all three were reduced in the DENV-2 infected saliva.

Two serine protease inhibitors (serpins) were identified via mass spectrometry and were reduced in the DENV-2 infected saliva. This finding mirrors that of Bonizzoni *et al*., who found a reduction in transcripts for serine proteases in DENV-infected *Aedes aegypti* midguts [17]. Both serpins are very similar, sharing 98% identity when compared at the amino acid level, therefore they are likely exhibit similar physiochemical properties. The protein identified as a salivary anti-coagulation factor Xa (FXa) serpin was reduced 4.8 fold (p ≤ 0.05) while the protein identified as serpin-4 was reduced 19.4 fold (p ≤ 0.05). The anti-coagulant salivary anti-FXa serpin was first identified by Stark and James as a ‘specific, reversible, noncompetitive, proteinaceous inhibitor of FXa’ [54,55]. This particular serpin is very similar to an *Ae. albopictus*-derived anti-FXa serpin termed ‘alboserpin’ [56]. Due to the detailed biochemical work done by Calvo *et al*. on alboserpin demonstrating binding affinities to heparin and phosolipid vesicles, interactions with phosphotidlycholine and phosphotydlethanolamine, and inhibiting FXa; the *Ae. aegypti* produced serpins may also share these properties.

The short form of aegyptin, also referred to as SAAG-4 by Boppana *et al*., was identified previously as being down-regulated in the salivary gland extract of *Ae. aegypti* infected with DENV-2 [24,57]. In agreement with the previous observation, this same aegyptin was found to be reduced in DENV-2 infected saliva by 14.1 fold (p ≤ 0.05). Beyond aegyptin’s role as an allergen, Calvo and others have thoroughly analyzed the physiological and biochemical capacities of the archetypal aegyptin molecule and found that it binds to collagen, inhibiting its interaction with platelet glycoprotein IV, integrin α2β1, and vonWillenbrand factor leading to an overall inhibition of coagulation [20,58-62]. It is important to note that this is not the only anti-coagulant protein reduced in the saliva of DENV-infected *Ae. aegypti*.

With three known salivary proteins related to the adenosine degrading complex of *Ae. aegypti* reduced in the DENV-2 infected saliva, along with a novel low density lipoprotein receptor that may be involved in the reduction of pain, it would appear that there is a trend towards the greater likelihood of both mast cell degranulation and pain perception compared to the same volume of uninfected mosquito saliva. Considering that the prevention of detection during feeding is a priority for *Ae. aegypti*, due to the fact that it possesses redundant machinery to remove adenosine, any reduction in expression of salivary proteins involved in that process could alert the host to the feeding attempt and lead to feeding interruptions causing failure to reach repletion. When combined with a reduction in salivary proteins involved in the prevention of clotting, such as the two serpins and the short form of aegyptin, yet another feeding pressure would be placed upon the infected mosquito compared to an uninfected one. If the saliva of an infected mosquito contains less anti-hemostatics on a per unit volume basis, as suggested from our data, there would be a greater chance of clot formation occurring during feeding which could also lead to an interruption in feeding with a failure to reach repletion. These two mechanisms which would hinder an infected mosquito during feeding could be the physiological basis for the interruptions or delays in feeding by DENV-infected mosquitoes reported by Platt *et al*. and Maciel-de-Freitas *et al*. [63,64].

These changes in salivary composition led us to revise the method of calculating vectorial capacity- a measure of transmission potential- of *Ae. aegypti*, a significant modifier of which is the biting rate of a vector on the pathogen-relevant vertebrate population (a) [65,66]. Typical formulations of the equation assume that the biting rate of mosquitoes is unchanged, regardless of infection status. However, the reduction in specific proteins identified in this research, combined with the changes in feeding behavior of DENV-infected mosquitoes seen by others, would indicate that this assumption may be violated in nature and would have consequences for transmission [63,64,67]. Thus, a separate parameter of a_INF_ to account for this alteration in vector biting rate due to infection status is appropriate. Indeed, the transmission differences due to the value of this new parameter a_INF_ are not inconsequential. Further, the reduction of these particular proteins may lead to 1) difficultly in immediate blood-meal acquisition and thus, additional probing by a foraging mosquito and 2) vertebrate host interruption of feeding by the mosquito. The combination of these two things could enhance the transmission success of DENV by 1) additional deposition of virus due to increased probing or 2) additional transmission events to a new vertebrate host due to probing interruption. The magnitude of this transmission success probability will require further experimental investigation, but given the estimated effects of this measure, this would merit investigation (Figure 2).

Finally, the remaining protein significantly reduced in the DENV-2 infected saliva 7.7 fold (p ≤ 0.05) was the hypothetical protein known through VectorBase.org as AAEL000732 [33]. Using cDART, no known conserved domains were identified and VectorBase.org has no information on suspected function. It would appear that the role of this protein in the saliva of *Ae. aegypti* remains to be determined.

## Conclusions

In summary, our findings indicate that DENV-2 infection alters the expression of various salivary proteins in *Ae. aegypti*, in particular proteins involved in anti-hemostatic and pain-reducing capacities. These changes may confer a fitness advantage upon the virus by enhancing viral establishment in the vertebrate or by increasing the number of transmission events. While this work is an important beginning, much remains to be characterized. In particular, the exact roles these salivary components have at the bite site within the context of viral deposition remains to be detailed. Restoration of reduced proteins and the resulting viral dynamics and host responses are currently being investigated and will likely be of use for vaccine development, treatment options, and a better understanding of the role of these critical vector components in arboviral transmission.

## Competing interests

The authors declare that they have no competing interests.

## Authors’ contributions

DMC designed the experiment, infected the mosquitoes, collected and purified saliva, performed 2-D gel electrophoresis, image analysis, data analysis, and contributed to the manuscript. RCC created the viral stocks, infected the mosquitoes, contributed to the manuscript, and performed statistical analysis and mathematic modeling. MKM assisted with data analysis and contributed to the manuscript. AMJ collected and purified saliva, extracted RNA and performed qRT-PCR for infection verification. BLL assisted with data analysis and contributed to the manuscript. CNM designed the experiment, assisted with data analysis, and contributed to the manuscript. All authors have read and approved the final version of the manuscript.

## Supplementary Material

Additional file 1 **SI1.** Description of parameters and equations used to model impacts of altered mosquito salivary expectorate on the vectorial capacity of *Ae. aegypti* mosquitoes. Click here for file

Additional file 2 **Mass_Spec_Supplement.** Excel formatted spreadsheet with additional information obtained from mass spectrometry analysis. Click here for file
