# Supplementary material for: Infection with dengue-2 virus alters proteins in naturally expectorated saliva of Aedes aegypti mosquitoes
Source: Parasit Vectors. 2014 May 30;7:252. doi: 10.1186/1756-3305-7-252 (PMC4057903; doi:10.1186/1756-3305-7-252)
Supplement: Additional file 1 — SI1. Description of parameters and equations used to model impacts of altered mosquito salivary expectorate on the vectorial capacity of Ae. aegypti mosquitoes. [file 1756-3305-7-252-S1.docx]

**Additional file 1**

Baseline vectorial capacity as in [[1](#_ENREF_1)] and with baseline value of t:

VC_base_ = $\frac{ma^{2}bp^{N}t}{-\ln p}$

Where

- m = mosquito density relative to humans = 1.9 [[2](#_ENREF_2)]
- a = daily biting rate (on relevant vertebrates) = .63 [[3](#_ENREF_3)]
- b = vector competence (ability for a mosquito to support replication of a pathogen) = .44 [[4](#_ENREF_4)]
- p = mosquito lifespan = .91 [[5](#_ENREF_5)]
- N = extrinsic incubation period (time in days it takes for a mosquito to be exposed to a pathogen and become subsequently infectious for the pathogen) = 9 days [[4](#_ENREF_4)]
- t = probability of transmission success due to altered salivar protein expression = .5 [[6](#_ENREF_6)]

Modified vectorial capacity with consideration for salivary protein alteration:

VC_mod_ = $\frac{ma\boldsymbol{a}_{\boldsymbol{INF}}bp^{N}\boldsymbol{t}}{-\ln p}$

Where

- a_INF_ = biting rate of infected mosquitoes due to protein alteration by DENV = [.88-1.63]
- t = probability of transmission success due to altered salivary protein expression = [.5-1]

Differences in vectorial capacity due to altered salivary protein expression:

ΔVC = VC_base_ - VC_mod_

The reduction in anti-hemostatics and/or pain inhibitory salivary components could lead to incomplete or interrupted feeding attempts by an infected mosquito, thereby leading to another feeding attempt on another host. This action modifies the parameter a, which is used to calculate VC, often based on models of ordinary differential equations, but which has an established form as in [1]. We have included an additional parameter, a_INF_, to account for the potential increased biting rate of infected mosquitoes compared to that of uninfected mosquitoes, as well as interrogated a range of transmission success probabilities to account for the potential enhancement of this probability, which has been shown to be imperfect [6].

References

1. Reisen WK: **Estimation of Vectorial Capacity: Introduction**. *Bulletin of the Society for Vector Ecology* 1989, **14**(1):39-40.

2. Jeffery JA, Thi Yen N, Nam VS, Nghia le T, Hoffmann AA, Kay BH, Ryan PA: **Characterizing the Aedes aegypti population in a Vietnamese village in preparation for a Wolbachia-based mosquito control strategy to eliminate dengue**. *PLoS neglected tropical diseases* 2009, **3**(11):e552.

3. Scott TW, Amerasinghe PH, Morrison AC, Lorenz LH, Clark GG, Strickman D, Kittayapong P, Edman JD: **Longitudinal studies of Aedes aegypti (Diptera: Culicidae) in Thailand and Puerto Rico: blood feeding frequency**. *Journal of medical entomology* 2000, **37**(1):89-101.

4. Christofferson RC, Mores CN: **Estimating the magnitude and direction of altered arbovirus transmission due to viral phenotype**. *PloS one* 2011, **6**(1):e16298.

5. Harrington LC, Buonaccorsi JP, Edman JD, Costero A, Kittayapong P, Clark GG, Scott TW: **Analysis of survival of young and old Aedes aegypti (Diptera: Culicidac) from Puerto Rico and Thailand**. *Journal of medical entomology* 2001, **38**(4):537-547.

6. Siler JF, Hall MW, Hitchens AP: **Dengue: its history, epidemiology, mechanism of transmission, etiology, clinical manifestations, immunity, and prevention**. Manila,: Bureau of Printing; 1926.
